# Composite Match Index with Application of Interior Deformation Field Measurement from Magnetic Resonance Volumetric Images of Human Tissues

**DOI:** 10.1155/2012/135204

**Published:** 2012-09-06

**Authors:** Penglin Zhang, Xubing Zhang, Jiangping Chen

**Affiliations:** ^1^School of Remote Sensing and Information Engineering, Wuhan University, Wuhan 430079, China; ^2^College of Mathematics and Computer Science, Wuhan Textile University, Wuhan 430073, China

## Abstract

Whereas a variety of different feature-point matching approaches have been reported in computer vision, few feature-point matching approaches employed in images from nonrigid, nonuniform human tissues have been reported. The present work is concerned with interior deformation field measurement of complex human tissues from three-dimensional magnetic resonance (MR) volumetric images. To improve the reliability of matching results, this paper proposes composite match index (CMI) as the foundation of multimethod fusion methods to increase the reliability of these various methods. Thereinto, we discuss the definition, components, and weight determination of CMI. To test the validity of the proposed approach, it is applied to actual MR volumetric images obtained from a volunteer's calf. The main result is consistent with the actual condition.

## 1. Introduction

The physical property is the base of the biological simulation, computer-assisted medical applications, such as clinical diagnosis, and surgical simulation, surgical planning. And estimation of internal deformation field or deformation motion for the biological tissues plays a very significant role in physical parameters estimation. Thus, measuring the internal deformation field of biological tissues is becoming the focus research. Magnetic resonance (MR) imaging (MRI) provides superb anatomic images with excellent spatial resolution and contrasts among soft tissues; thus, it is widely used in computer-assisted medical applications, such as clinical diagnosis, surgery simulation, operation planning, and evaluation of physical characteristics of biological tissues. Increasing number of researchers in medical simulation and medical virtual reality focus on the interior deformation field or motion measurement of biological tissues from MR volumetric images, and it has become one of the significant branches of medical image analysis. Generally, approaches for estimating the deformation of MR volumetric images can be classified into two typical types: elastic deformation model-based and feature matching-based methods.

The elastic deformation model-based method can be classified into either parametric or geometric active models [1]. To obtain the deformation information of an object, the parametric active contours, also called snakes, try to minimize a defined cost function so that the function deforms a given initial contour toward the boundary of the object. This method was first introduced by Kass et al. in 1987 [2] and subsequently developed and used by Lang et al. [3], Cho and Benkeser [4], and Matuszewski et al. [5] to estimate deformation motion of nonrigid objects. In the geometric active model [1, 6–8], the curve and the surface of an object are first detected. Then, the deformation propagation of the curve and the surface is used to track the motion. However, irrespective of what elastic deformation models are employed, disadvantages exist in the deformation estimation; for example, the parametric active model cannot handle changes in the topology of the evolving contours when deformation is performed directly, and often, heuristic topology handling procedures are used [8]. In the geometric active model, when contrast is poor and boundaries are not clear or are continuous in the images, the contours tend to leak through the boundary [9]. The tagged images must have a regular grid pattern in the imaging plane because if the number of tagged points is low, the measurement accuracy would be poor. More important than the former two aspects, regardless of what elastic deformation models are used, they can only handle the deformation at the boundary of nonrigid objects and not the interior deformation.

In recent years, researchers have been increasingly concerned on approaches for matching of nonrigid feature points. Typically, thin-plate spline-robust point matching (RPM) is a famous algorithm for matching non-rigid feature points, which can estimate the joint correspondence and non-rigid transformations between two differently sized point sets. However, optimal processing of the energy function utilized in Chui's method may be trapped in bad local minima [10]. Zheng proposed the RPM-local neighborhood structure (LNS) method of matching non-rigid feature points, based on the supposition that relative distances and orientations among feature points in a neighborhood would be preserved [11]. Lee improved the LNS and presented the topology preserving relaxation labeling (TPRL) algorithm. In the TPRL method, log distance and polar angle bins are utilized to capture the coarse location information of the feature points in a neighborhood. Using shape contexts, Belongie proposed a non-rigid point matching method. In this method, every feature point is represented by a histogram descriptor of the distance and orientation between this feature point and its neighbor feature points [12]. In addition, some other useful methods were also proposed for feature-point matching, such as the coherent point drift matching method of non-rigid points [13, 14] and the preservation of local geometrical characteristics [15]. In these methods, a novel objective function is defined to preserve local image-to-image affine transformations across correspondence. In general, some unsolved problems are involved in the aforementioned matching methods of non-rigid points; for example, the optimal processing of the energy function could be trapped in bad local minima, the topology of the neighboring feature points is not always preserved well, and so on. Most importantly, in these methods, useful information of the feature point is considered singly and lacks a comprehensive approach, which can mix up with the useful and significant information in the point matching of deformation measurement.

Therefore, to improve further the proposed feature-matching-based approach and improve the robustness of the matching result, this paper proposes a composite match index (CMI). In Section 2, we introduce some previous work, in Section 3, we describe the concept and definition of CMI, and Section 4 introduces the CMI application on feature matching of image pairs from non-rigid objects. In Section 5, examples and preliminary experimental results are given, and discussion and conclusions are presented in the final section.

## 2. Previous Work

Feature matching plays a significant role in human visual perception, recognition, and computer vision. In medical imaging, most existing feature matching-based research has focused on non-rigid registration and internal deformation field measurement. The general idea of these works is first to extract enough feature points or markers from medical images acquired from non-rigid objects on natural and deformed states, respectively. Next, the feature matching algorithm is applied on extracted feature-point sets to establish robust corresponding pairs. Finally, corresponding pairs are used as control points in non-rigid registration and are used to calculate sparse deformation fields in internal deformation field measurements. Therefore, finding robust corresponding pairs is a vital problem in the present work. We surveyed existing works on feature-point matching in computer vision. Relaxation is a valid technique to disambiguate matches and improve the robustness of matches. Finding a globally optimal or reasonably good suboptimal solution in relaxation is a difficult task, and such matching techniques in non-rigid medical image processing have been rarely addressed. However, a potential advantage is that harder matching problems can be solved using global optimization techniques.

Papademetris et al. [16] presented a method for the integration of feature and intensity information for non-rigid registration. In this case, a distance-based robust point matching framework was proposed to estimate feature-point correspondences. A disadvantage of the algorithm is that it estimates transformation using weighted least squares, which affects the strength of matching.

Zhang et al. [17] introduced a feature matching-based algorithm and considered the problem of 2D deformation field measurement as an example. Matching strengths are measured using correlation and relative distance between two feature points. Relaxation by the optimization algorithm is deductive of the function of matching strength. In later research [18], after slight revision, the algorithm was extended to a 3D situation because the intensity in a magnetic resonance (MR) image is the information of tissue mapped on an image. Thus, the correlation intensity of regions between two points in matching and relaxation can effectively use the properties of tissue.

The work [19] proposed a local geometric preserving algorithm to find corresponding feature pairs from given feature points set in MR volumes acquired from an object on natural and deformed states, respectively. The main contribution of the algorithm to feature matching is that for a non-rigid tissue, when an outside force is applied on it, the deformation magnitude and orientation are different in different regions. However, for a local region on the object, the difference is actually very slight and can sometimes be ignored.

Problems in image feature-point matching remain as great challenges for medical image processing. Thus, the accuracy of feature matching needs to be further improved. Typically, single factors, such as intensity and distance, are effective in matching algorithm for specific areas. However, total accuracy cannot be improved. The integration of multifactors to form a composite approach can make use of the advantages of each factor to improve total accuracy. The present work proposes a composite framework that can pose multicomponents in a single cost function with associated weights to find corresponding feature pairs.

## 3. CMI

 CMI-based feature-point matching approach was proposed to address the fusion of different operator types and to improve the reliability of results from single operators. Here, CMI is a scalar quantity that describes the matching possibility of point pairs. Let

(1) $$\begin{array}{c} c_{i}={\left[c_{i,1},c_{i,2},\ldots ,c_{i,k}\right]}^{T},\\ w={\left[w_{1},w_{2},\ldots ,w_{k}\right]}^{T} \end{array}$$

be the vector of component value and its corresponding weight, respectively. Then, according to the linear weighting method, CMI is defined as

(2) $$\begin{array}{c} \xi_{i}^{t+1}=\sum_{k=1}^{K}w_{k}^{t+1}\cdot c_{i,k} \end{array}$$

subject to

(3) $$\begin{array}{c} \sum_{k=1}^{K}w_{k}=1, \end{array}$$

where *ξ*_*i*_ represents the CMI of the *i*-th pair, *c*_*k*_ represents the value of the *k*th component consisting of the CMI, *w*_*k*_ represents the weight of the *k*th component, and *K* is the number of components in the CMI. Here, component is a factor that can be used to evaluate feature-point pair similarities. Weight *w*_*k*_ is used to measure the significance of a component for CMI. Various weighting methods have been reported for different research fields. In this case, to consider the independence of each component, the correlation weighted method is used to determine the weight of each component. Let **r** = [*r*_1,1_, *r*_1,2_,…, *r*_1,*k*_]^*T*^ be the correlation vector consisting of correlation score of the component 0 and *k*. Then, the weight of the *t* + 1 time *w*_*k*_^*t*+1^ is defined by

(4) $$\begin{array}{c} w_{k}^{t+1}=\frac{\left|r_{1,k}^{t}\right|}{\sum_{k=1}^{K}\left|r_{1,k}^{t}\right|} \end{array}$$

with

(5) $$\begin{array}{c} r_{1,k}=\frac{\sum_{i=1}^{N}\left(c_{i,1}-{\overset{-}{c}}_{1}\right)\left(c_{i,k}-{\overset{-}{c}}_{k}\right)}{\sqrt{\sum_{i=1}^{N}{\left(c_{i,1}-{\overset{-}{c}}_{1}\right)}^{2}}\sqrt{\sum_{i=1}^{N}{\left(c_{i,k}-{\overset{-}{c}}_{k}\right)}^{2}}},\;{\overset{-}{c}}_{k}=\frac{1}{N}\sum_{i=1}^{N}c_{i,k}, \end{array}$$

where *c*_*i*,*k*_ is the value of the *i*-th feature-point pair and *N* is the total number of match pairs in the potential matching set obtained at time *t*.

Since feature-point pairs within the potential matching set obtained at time *t* are used as samples to compute the weight *w*_*k*_^*t*+1^ of the *k*th component in *t* + 1 times iteration, the pairs in potential matching set are different at each time. Thus, *w*_*k*_ values are also different at different times, keeping iterations in the matching process.

CMI is an effective way to fuse multifeature matching algorithm. CMI takes full advantage of all the considered factors to generate a more robust feature matching approach and obtain more accurate matching results. Thus, the feature-point matching algorithm, which decides the strength of matching via a similarity judge function, can theoretically be integrated as a CMI component. In this case, the local geometric persistence (LGP), local intensity similarity (LIS), and local correlation score (LCS) between regions around participants are selected as the components to compute the CMI of a match pair (**p**_*u*,*i*_, **p**_*v*,*i*_) and demonstrate the validity of CMI. The following section will discuss how to compute LCS, LGP, and LIS.

For convenient descriptions, several definitions are first clarified as follows

1. Initial feature set **p**_*u*_, feature-point set extracted from the MR volume acquired from the object at a natural state.
2. Deformed feature set **p**_*v*_, feature-point set extracted from the MR volume acquired from the object at a deformed state.
3. PMS, a potential feature match set composed of a match pair (**p**_*u*,*i*_, **p**_*v*,*i*_) if and only if the best match of **p**_*u*,*i*_ is **p**_*v*,*i*_ and conversely **p**_*u*,*i*_ is also the best match of **p**_*v*,*i*_.
4. **p**_*u*,*i*_ represents the feature point *i* in the initial feature set, and **p**_*v*,*i*_ represents the feature point *i* in the deformed feature set.

### 3.1. LGP

 Let **c**_*u*_ and **c**_*v*_ be the moment center computed using the initial feature set and its mapping in the deformed feature set, respectively, let **p**_*u*,*i*_ be the *i*th point in the initial feature set, and let the mapping in the deformed feature set be **p**_*v*,*i*_. Based on the consistent deformation in a local region, the distance ratio of a potential match pair in a local region far from their moment center is equivalent and thus yields *μ*_*i*,*j*_:

(6) $$\begin{array}{c} \mu_{i,j}=\frac{d\left(p_{u,i,j},c_{u}\right)}{d\left(p_{v,i,j},c_{v}\right)}\approx \frac{1}{J}\sum_{j=1}^{J}\frac{d\left(p_{u,i,j},c_{u}\right)}{d\left(p_{v,i,j},c_{v}\right)}, \end{array}$$

where *μ*_*i*,*j*_ is the distance ratio of the *j*th potential match pair (**p**_*u*,*i*,*j*_, **p**_*v*,*i*,*j*_) in the local region around pair (**p**_*u*,*i*_, **p**_*v*,*i*_), *d*(**p**_*u*,*i*,*j*_, **c**_*u*_) is the Euclidian distance between **p**_*u*,*i*,*j*_ and **c**_*u*_, *d*(**p**_*v*,*i*,*j*_, **c**_*v*_) is the Euclidian distance between **p**_*v*,*i*,*j*_ and **c**_*v*_, and *J* is the number of potential match pairs in the local region. Ideally, *μ*_*i*,*j*_ should be a constant in the local region.

Moreover, **d**_*u*,*i*_ = [*d*(**p**_*u*,*i*,1_, **c**_*u*_), *d*(**p**_*u*,*i*,2_, **c**_*u*_),…, *d*(**p**_*u*,*i*,*J*_, **c**_*u*_)]^*T*^ and **d**_*v*,*i*_ = [*d*(**p**_*v*,*i*,1_, **c**_*v*_), *d*(**p**_*v*,*i*,2_, **c**_*v*_),…, *d*(**p**_*v*,*i*,*J*_, **c**_*v*_)]^*T*^ are the distance sets of the potential pairs within a local region around pair (**p**_*u*,*i*_, **p**_*v*,*i*_), respectively. Based on the definition of mathematical expectation, we yield

(7) E(du,i)=1J∑j=1Jd(pu,i,j,cu),E(dv,i)=1J∑j=1Jd(pv,i,j,cv).

Thus, if **p**_*v*,*i*_ in the deformed feature set is the best match of a given feature **p**_*u*,*i*_ in the initial feature set, then, the geometric deformation of potential match pair (**p**_*u*,*i*,*j*_, **p**_*v*,*i*,*j*_) within a local region around pair (**p**_*u*,*i*_, **p**_*v*,*i*_) is defined as

(8) $$\begin{array}{c} g_{i,j}=\left|\mu_{i,j}-\eta_{i}\right| \end{array}$$

subject to

(9) $$\begin{array}{c} \eta_{i}=\frac{E\left(d_{u,i}\right)}{E\left(d_{v,i}\right)}. \end{array}$$

In a small local region, all the *g*_*i*,*j*_(*j* = 1,2,…, *J*) should be approximately identical and go to zero; the smaller the value of *g*_*i*,*j*_, the better the geometric persistence of a potential match pair (**p**_*u*,*i*_, **p**_*v*,*i*_). This is called geometric persistence in this case. Thus, the impact factor of the *j*-th feature pair for the LGP within a small local region is

(10) $$\begin{array}{c} \lambda_{i,j}=\frac{1.0}{1.0+g_{i,j}}. \end{array}$$

The geometric property within a local region is approximately consistent in the initial and deformed states. If a pair is the best match for each other, then the correlation of potential matches within a local region around the pair must be a strong one. The correlated score *gc*(**p**_*u*,*i*_, **p**_*v*,*i*_) of the geometric persistence of PMS in a small local region around (**p**_*u*,*i*_, **p**_*v*,*i*_) can represent the LGP of feature pair (**p**_*u*,*i*_, **p**_*v*,*i*_), specifically:

(11) gc(pu,i,pv,i)=∑j=1Jλi,j(d(pu,i,j,cu)−E(du,i))∑j=1J(d(pu,i,j,cu)−E(du,i))2         ×(d(pv,i,j,cv)−E(dv,i))∑j=1J(d(pv,i,j,cv)−E(dv,i))2,

where *J* is the number of potential matches within a local region. In (11), if *λ*_*i*,*j*_ is large, the pair (**p**_*u*,*i*,*j*_, **p**_*v*,*i*,*j*_) may be a strong match pair; thus, its weight must also be large. In addition, the value range of *gc*(**p**_*u*,*i*_, **p**_*v*,*i*_) should be [−1,1]. Normalizing *gc*(**p**_*u*,*i*_, **p**_*v*,*i*_) yields normalized LGP as

(12) $$\begin{array}{c} N_{gc}\left(p_{u,i},p_{v,i}\right)=\frac{1+gc\left(p_{u,i},p_{v,i}\right)}{2}. \end{array}$$

### 3.2. LIS

LIS is used to describe the intensity difference between regions around a feature-point pair in the initial and deformed volumes. As mentioned earlier, the tissue within a local region is the same in the initial and deformed states. Thus, based on the MRI principle, the intensity difference is small. The inner product between two regions has the same properties with the invariance of rotation, zoom in, and zoom out. The normalized inner product between regions around (**p**_*u*,*i*_, **p**_*v*,*i*_) is adopted to define the similarity of two regions. Thus,

(13) $$\begin{array}{c} \text{lis}\left(p_{u,i},p_{v,i}\right)=\frac{X_{u,i}^{⊤}X_{v,i}}{||X_{u,i}||\cdot ||X_{v,i}||}, \end{array}$$

where *X*_*u*,*i*_ is the region in the initial volume centered at feature **p**_*u*,*i*_ and *X*_*v*,*i*_ is the mapping region of *X*_*u*,*i*_ centered at feature **p**_*v*,*i*_.

### 3.3. LCS

Let *I*(**p**_*u*,*i*,*m*_) and *I*(**p**_*v*,*i*,*m*_) be the intensity of the *m*-th voxel within the region centered at **p**_*u*,*i*_ and **p**_*v*,*i*_ in the initial and deformed MR volumes, respectively. Let *𝒪* be the local cubic region with a size of *w* × *h* × *l*. The local correlation score between local cubic regions around feature **p**_*u*,*i*_ in the initial MR volume and its candidate match feature **p**_*v*,*i*_ in the deformed MR volume is defined as

(14) $$\begin{array}{c} \text{lcs}\left(p_{u,i,m},p_{v,i,m}\right)=\frac{\sum_{m=1}^{M}\left(I\left(p_{u,i,m}\right)-{\overset{-}{a}}_{u,i}\right)\left(I\left(p_{v,i,m}\right)-{\overset{-}{a}}_{v,i}\right)}{M\sqrt{\sigma^{2}\left(I\left(p_{u,i,m}\right)\right)\cdot \sigma^{2}\left(I\left(p_{v,i,m}\right)\right)}}, \end{array}$$

where

(15) $$\begin{array}{c} M=w\times h\times l,\\ {\overset{-}{a}}_{u,i}=\frac{1}{M}\sum_{m=1}^{M}I\left(p_{u,i,m}\right),\;\;{\overset{-}{a}}_{v,i}=\frac{1}{M}\sum_{m=1}^{M}I\left(p_{v,i,m}\right). \end{array}$$

Here, *σ* ^2^(*I*(**p**_*u*,*i*,*m*_)) and *σ* ^2^(*I*(**p**_*v*,*i*,*m*_)) are the standard derivation of the local region *𝒪* around feature **p**_*u*,*i*_ and **p**_*v*,*i*_, respectively. They are given by

(16) σ2(I(pu,i,m))=∑m=1M(I(pu,i,m)−a−u,i)2M,σ2(I(pv,i,m))=∑m=1M(I(pv,i,m)−a−v,i)2M,

where ${\overset{-}{a}}_{u,i}$ and ${\overset{-}{a}}_{v,i}$ are the averaged intensity in the neighborhood of feature **p**_*u*,*i*_ and **p**_*v*,*i*_, respectively.

## 4. Application in Feature Matching

This section describes the measurement of internal deformation fields using CMI. First, the cost function is given to obtain optimal feature pairs iteratively. Then, the actual feature matching algorithm is described. Finally, the internal deformation fields are measured using optimal feature pairs. 

### 4.1. Cost Function

 CMI is an index that measures the strength between a given feature and its candidate matches in feature matching. In theory, for a given reference feature, its potential match must have the strongest CMI among all the candidates. Thus, for an optimal potential matching set, its whole CMI will also be the strongest. Based on this idea, we yield

(17) $$\begin{array}{c} S^{t}=\frac{1}{N}\sum_{i=1}^{N}\xi_{i}^{t}, \end{array}$$

where *S* is the cost function in iteration and *N* represents the total number of match pairs in the PMS obtained at time *t*.

### 4.2. Actual Matching Algorithm

The objective of the feature matching algorithm is to obtain an optimal PMS ultimately. The idea of PMS optimization is to maximize the aforementioned cost function *S* iteratively. In each iterative step, the current PMS strength is evaluated by all candidate matches within PMS using the defined cost function *S*. The iterative steps will stop until *S* no longer increases or is subjected to stop conditions. Specifically, the inputs are two feature-point sets obtained from MR volumetric images of an object under natural and deformed states, respectively. The output is an optimal PMS. The specific process of the algorithm is summarized as follows.

(0) Compute LCS and LIS. For each given pair (**p**_*u*,*i*_, **p**_*v*,*i*_) consisting of features in initial and deformed volumes, we use a local region (size of 9 × 9 × 3 in this case) centered at features to compute LCS and LIS according to (14) and (13), respectively.
(1) Form initial PMS. The LCS is used as the initial CMI of each match pair in the step of initial PMS formation. In other words, LCS is the only criterion of this step.
(2) Compute LGP. For each given pair (**p**_*u*,*i*_, **p**_*v*,*i*_), we first search for neighbor potential matches within a small window (size of 17 × 17 × 3 in this case) centered at **p**_*u*,*i*_. The potential matches contained within the window are participants in the LGP computation using the approach in Section 3.1.
(3) Compute **w**. Compute the weight for each CMI component using potential matches in current PMS as samples. The specific computing method can be seen in (4).
(4) Update the CMI of each pair. For each given pair (**p**_*u*,*i*_, **p**_*v*,*i*_), its corresponding CMI is updated through the weighting sum of the components LCS, LIS, and LCP, which are computed in (0) and (2).
(5) Form PMS and compute the cost function *S*. The updated CMI of each pair forms new PMS. The cost function in (17) is then computed using potential matches in the current PMS.
(6) Repeat (2) to (5) until *S* no longer increases.
(7) Return the current PMS.

Although candidate sets LCS and LIS of each pair are constant, PMS is dynamic because of the varying LGP and **w** of the component at *t* + 1 times iteration. Thus, the match strength index of CMI is varied. Dynamic cost function will move potential matches into or out of the PMS. The best candidate of a feature-point may also change.

### 4.3. Measuring Density Deformation Fields

 After obtaining the optimal PMS, the internal density deformation fields of non-rigid objects are then obtained. In this study, the method proposed in our previous work [20] is used to obtain the internal density deformation fields. In summary, the internal density deformation fields are interpolated by sparse deformation fields using a finite element model. In detail, the magnitude of the sparse deformation field is first computed by its corresponding pair in PMS using Euclidian distance. The start and end points of a field direction are defined by the points of the corresponding pair. Next, a non-rigid object is reconstructed using tetrahedra, whose nodes are points in the PMS. The density deformation fields can then be interpolated using the finite element method.

Let **P** be an arbitrary volume voxel at **x** = (*x*, *y*, *z*) within a tetrahedron ◊**P**_*i*_ **P**_*j*_ **P**_*k*_ **P**_*l*_ consisting of nodal points **P**_*i*_, **P**_*j*_, **P**_*k*_, and **P**_*l*_. Its displacement may be approximated by weighting the finite element node displacements **u**_*i*,*j*,*k*,*l*_(**x**) using their shape function [20]:

(18) u(x)=ui(x)Ni,j,k,l(x)+uj(x)Nj,k,l,i(x)    +uk(x)Nk,l,i,j(x)+ul(x)Nl,i,j,k(x),

where **u**_*i*_(**x**) is the displacement of nodal *i*, and the shape function *N*_*i*,*j*,*k*,*l*_(**x**) on tetrahedron ◊**P**_*i*_ **P**_*j*_ **P**_*k*_ **P**_*l*_ is given by

(19) $$\begin{array}{c} N_{i,j,k,l}\left(x\right)=\frac{♦PP_{j}P_{k}P_{l}}{♦P_{i}P_{j}P_{k}P_{l}}, \end{array}$$

where *♦* **P** **P**_*j*_ **P**_*k*_ **P**_*l*_ and *♦* **P**_*i*_ **P**_*j*_ **P**_*k*_ **P**_*l*_ are the volume of tetrahedron ◊**P** **P**_*j*_ **P**_*k*_ **P**_*l*_ and ◊**P**_*i*_ **P**_*j*_ **P**_*k*_ **P**_*l*_, respectively.

## 5. Experiments and Results

Our approach consists of four steps: feature extraction, affine transformation, feature matching, and deformation field measurement. Extracting sufficient features from the initial and deformed volumes is necessary to find enough homologous feature pairs. In this study, high-curvature 3D points were preextracted as features from MR volumetric images. In this case, the two-dimensional Harris operator [21] was extended to a 3D operator by extracting features from the MR volumetric images [22].

Some experiments were designed to demonstrate the performance of the proposed approach. All experiments were performed using our own tool developed with Visual C++, which runs on Microsoft Windows XP. All described experimental results were obtained on a Lenovo Portable PC with a 2.20 GHz Intel(R) Core(TM) 2 Duo CPU T6600 and 4 GB of RAM.

**Figure 1:**
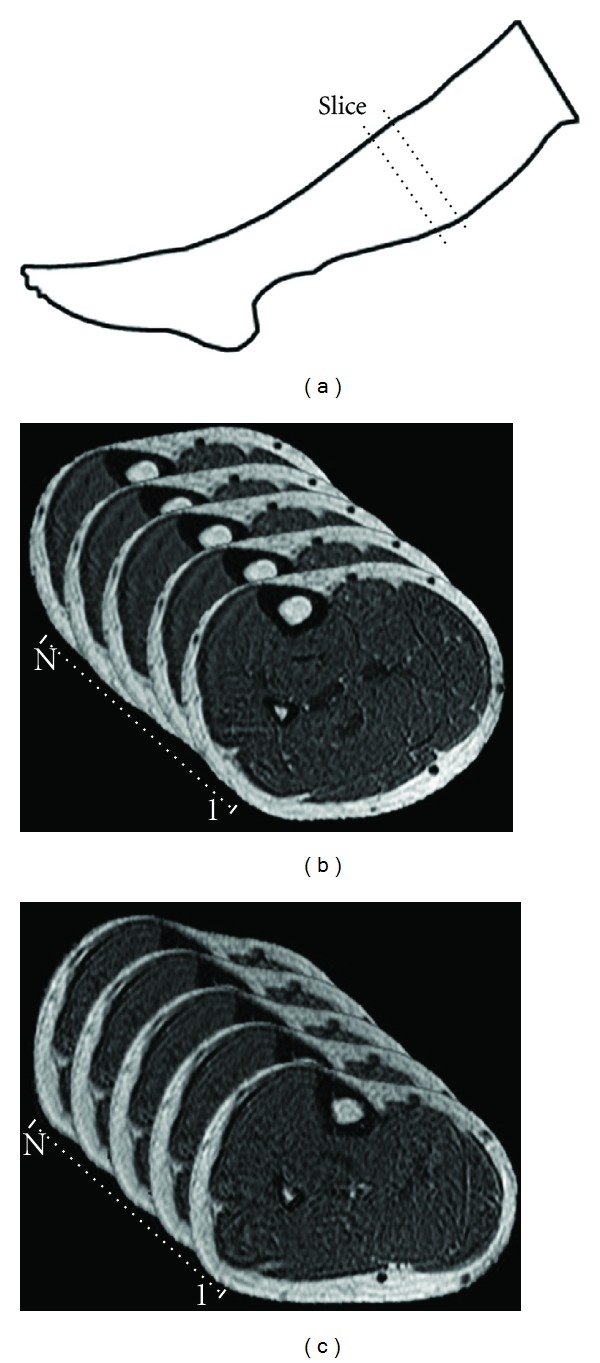
Acquired MR volumes. (a) Place of acquired volume; (b) MR slices in volume obtained at natural state; (c) MR slices in volume obtained at deformed state.

In the experiment, the MR images were acquired from a volunteer's calf (Figure 1(a)) using an MRI scanner at natural state and deformed states (initial and under forcing), respectively. In both cases, the FOV was 20 × 20 cm, and the slice gap was 2 mm. Some slices (Figures 1(b) and 1(c)) placed at the middle section of the calf were selected to form the MR volumes. As a result, initial and deformed volumes with size of 512 × 512 × 57 voxels were generated for the experiment.

**Figure 2:**
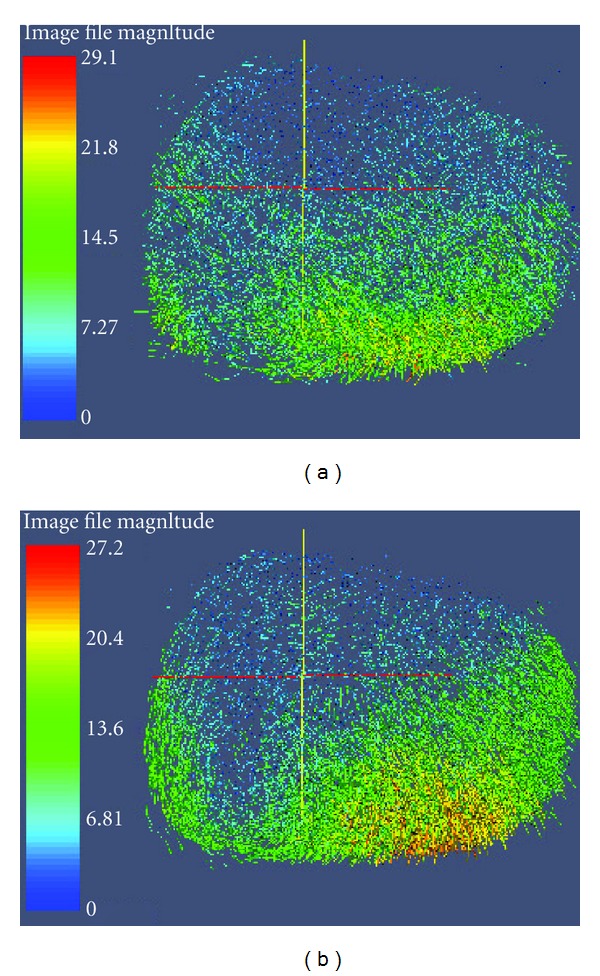
Density deformation fields. Deformation fields generated using PMS obtained using the (a) CMI-based feature match algorithm and the (b) RPFM algorithm).

First, 500 and 800 features were extracted from the volume acquired on the natural and deformed states, respectively. Next, the proposed CMI-based feature match approach was applied on the two feature-point sets to obtain the optimal PMS. As the result, a PMS with 245 potential match pairs was obtained. The sparse and density deformation fields were computed using the method mentioned in Section 4.3.  Figure 2 shows 50000 internal density deformation fields, with large deformation at the bottom of the calf. This result is consistent with the actual situation. 

 To prove the validity of the proposed CMI-based feature match algorithm, we compared it with a robust point feature matching (RPFM) algorithm proposed by Chen [23]. In the present study, we applied the RPFM algorithm to the same feature-point sets, which resulted in a PMS with 316 potential match pairs.

**Figure 3:**
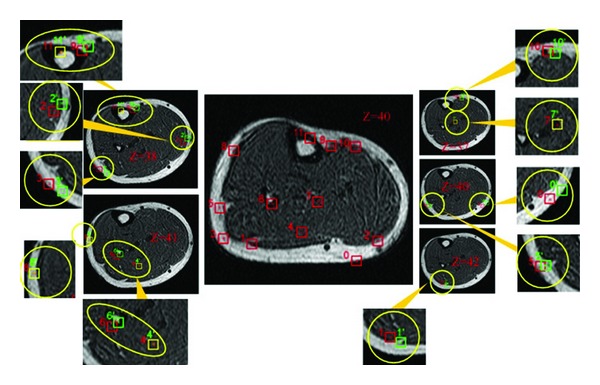
Reverse moving result of the landmarks using deformation fields measured through the CMI-based approach.

**Figure 4:**
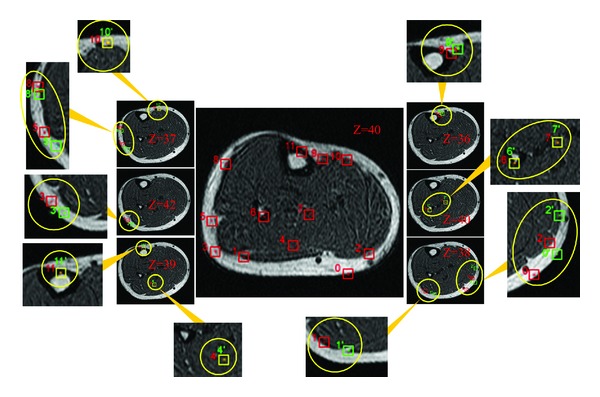
The reverse moving result of the landmarks using deformation fields measured through the RPFM approach.

We selected 12 landmarks in the slice (*z* = 40) of deformed MR volume to test the accuracy of the measured internal deformation fields, as shown in the middle picture of Figures 3 and 4. Then, the landmarks were subjected to reverse moving using the internal deformation fields measured through the CMI-based algorithm and RPFM algorithm. The results on the MR volume acquired at natural state were projected to check the accuracy of the deformation fields. Figures 3 and 4 show the reverse moving results of the landmarks.

In Figures 3 and 4, the center of each red rectangle in the middle picture (*z* = 40) gives the landmark position. Slices that lie on the left and right sides (the middle layer) give the reverse moving result of the landmarks and the *z* value of different slices, respectively. The outer layer is the zoom in for the reverse moving result of each landmark. In the middle and outer layers, the red rectangles represent the reverse moving position of the landmarks, the green rectangles are actual position of landmarks, and the yellow rectangles represent the reverse moving positions and actual position consistency. From Figures 3 and 4, we note the accuracy of the reverse moving position of landmarks using deformation fields calculated by PMS obtained using CMI-based approach obviously is higher than that of RPFM, that is, the reverse moving position of landmarks 0, 1, 2, 3, and 5.  Table 1 shows the quantitative accuracy of the reverse moving results of the landmarks using internal deformation fields obtained by PMS via CMI and RPFM. 

As shown in Table 1, regardless of the direction (i.e., *x*-, *y*-, and *z*-directions), the accuracy of the deformation fields measured through PMS obtained using the CMI-based approach is better than that using the RPFM algorithm.

The number of potential matches in optimal PMS obtained using the CMI-based feature matching algorithm is fewer than that of RPFM because the CMI-based approach is combined with the multifeatures in feature matching, whereas RPFM is a single-feature approach. In other words, the match requirements of CMI are stricter compared with those of RPFM. The reliability of optimal PMS obtained using the CMI-based algorithm is higher because it has more accurate deformation fields than the RPFM algorithm. This conclusion is supported by the reverse moving results of the landmarks.

## 6. Conclusions

In this work, a new method called CMI is presented for the integration of feature-based internal deformation field measurements. In general, feature match algorithms using a single property are highly accurate in specific aspects. However, the overall accuracy is limited because the full advantages of different properties in feature-point matching are not fully used. Fusion multialgorithms offer the use of advantages in algorithms to improve accuracy. Such a fusion is necessary for feature matching in non-rigid objects, where the improvement will be more obvious. In addition, the most advantage of the proposed approach is to provide a feasible option to integrate various feature matching algorithms. Each feature matching algorithm can act as the component of the CMI, and if the appropriate weight can be assigned to the component, then, one can obtain more reliable potential matches. Obviously, the effect of the component weight should also be considered. Thus, (1) investigating an approach to determine the appropriate weights should be the focus of future research; (2) the imaging mechanism of MRI should be further considered in component of the CMI to remove the aberrance of machine to improve the accuracy of feature-point matching as possible.

## Figures and Tables

**Table 1:** Comparison of the accuracy in different directions.

Approach	Error	Number of landmarks												RMSE
		0^#^	1^#^	2^#^	3^#^	4^#^	5^#^	6^#^	7^#^	8^#^	9^#^	10^#^	11^#^
	*x*-error	− 12	− 8	− 7	− 13	0	− 4	− 6	0	0	− 7	0	0	6.626965
CMI	*y*-error	8	− 6	7	− 7	0	1	6	0	0	5	0	0	4.654747
	*z*-error	0	− 2	2	2	− 1	0	− 1	3	− 1	2	3	2	1.848423

	*x*-error	− 21	− 23	− 9	− 12	0	− 11	0	0	− 1	6	0	0	10.61838
RPFM	*y*-error	20	− 8	26	− 10	0	− 13	0	0	− 7	4	0	0	11.08302
	*z*-error	2	2	2	− 2	1	3	0	0	3	4	3	1	2.254625

RMSE: root mean square error; *x*-error: error in the *x*-direction; *y*-error: error in the *y*-direction; *z*-error: *z*-error in the *z*-direction.
